# Farmed fish welfare during slaughter in Italy: survey on stunning and killing methods and indicators of unconsciousness

**DOI:** 10.3389/fvets.2023.1253151

**Published:** 2023-10-05

**Authors:** Gianfilippo Alessio Clemente, Clara Tolini, Andrea Boscarino, Valentina Lorenzi, Tania Lidia Dal Lago, Daniele Benedetti, Fabio Bellucci, Amedeo Manfrin, Angela Trocino, Sara Rota Nodari

**Affiliations:** ^1^Italian National Reference Center for Animal Welfare, Istituto Zooprofilattico Sperimentale della Lombardia e dell’Emilia Romagna ‘Bruno Ubertini’, Brescia, Italy; ^2^Istituto Zooprofilattico Sperimentale della Lombardia e dell’Emilia Romagna ‘Bruno Ubertini’, Brescia, Italy; ^3^Italian Ministry of Health, Rome, Italy; ^4^National Reference Laboratory for Crustacean Diseases, Istituto Zooprofilattico Sperimentale della Venezie, Legnaro, Italy; ^5^Department of Agronomy, Food Natural Resources Animals Environment, University of Padua, Legnaro, Italy; ^6^Department of Comparative Biomedicine and Food Science, University of Padua, Legnaro, Italy

**Keywords:** aquaculture, fish unconsciousness, thermal shock, electrical stunning, trout, seabream, seabass, sturgeon

## Abstract

Information on slaughter procedures for farmed fish in aquaculture is limited, both in Europe and in Italy, due to a general lack of field data. The aim of this study was to gather information on the procedures used to slaughter fish in Italy and to discuss them considering the WOAH and EFSA recommendations on fish welfare. Using a questionnaire survey, data were collected by official veterinarians in 64 slaughtering facilities where 20 different species of fish were slaughtered. The main species slaughtered were rainbow trout (*Oncorhynchus mykiss*; 29/64), followed by European sea bass (*Dicentrarchus labrax*; 21/64), sea bream (*Sparus aurata*; 21/64), Arctic char (*Salvelinus alpinus*; 14/64), European eel (*Anguilla anguilla*; 11/64), sturgeon (*Acipenser* spp; 11/64), common carp (*Cyprinus carpio*; 6/64), and brown trout (*Salmo trutta fario* L.; 5/64). The most applied stunning/killing methods were “asphyxia in ice/thermal shock” and “electric in water bath,” followed by “percussion,” “asphyxia in air,” and “electric dry system.” After the application of the method, the assessment of the fish level of unconsciousness was practiced in 72% of the facilities using more than one indicator, with “breathing” and “coordinated movements” the most practiced. The collected data showed a discrepancy between the available recommendations about the welfare of fish at slaughter and what is practiced in many production sites, but for many species precise recommendations are still not available.

## 1. Introduction

Aquaculture production has experienced significant growth over the past two decades (1–3), reaching 87.5 million tons in 2020 at a global level (2). This rapid expansion has led to considerable interest in welfare issues by scientists, consumers and policy makers. The number of scientific studies claiming that fish are sentient animals, able to perceive emotions and thus of experiencing fear, psychological stress, and pain is increasing, as recently reviewed (4). Consumers demand information about the origins of food products and the conditions under which farmed animals are kept (5). Animalist groups claim more than half of the consumers are aware that fish are capable of experiencing pain and that the current protection of fish welfare is insufficient if compared to the other farmed species (6). In fact, despite that the number of farmed and slaughtered fish for human consumption is largely higher than that of farmed mammals (approximately 2.5-fold) (3, 7), the welfare of farmed fish is inadequately protected by the European legislation (3, 6). Specifically for the protection of animals at the time of killing (EC Reg. 1099/2009) (8) fish are included only within the general framework of basic principles. Only the Article 3 (1) of the general provision is applicable for fish, according to which animals for slaughter must be protected from avoidable pain, distress or suffering. Unlike for other species, the methods for fish stunning and killing are not defined. Actually, in the absence of scientific updates and EU rules, Article 27 (1) allows Member States to maintain or adopt national rules regarding the protection of fish at the time of killing (8). However, only a few Member States have implemented national laws which refer only to a few species (e.g., salmon) (9). In order to cope with these legislative gaps, the Farm to Fork strategy of the European Green Deal commits to review and update European animal welfare legislation (10) and for the slaughter of fish, in particular, it is intended to add more guidelines for the main farmed species and to revise welfare assessment criteria that are often inapplicable in the field (10).

In Europe, approximately 50 species of fish are reared for human consumption purpose. The most farmed species is rainbow trout (*Oncorhynchus mykiss*), followed by European seabass (*Dicentrarchus labrax*), gilthead seabream (*Sparus aurata*), common carp (*Cyprinus carpio*), and atlantic salmon (*Salmo salar*) (2, 11). Currently, farmed fish can be subjected to a range of different stunning/killing methods at slaughter. Many of these methods present relevant welfare problems, since they expose fish to prolonged suffering and pain before death (12–19).

Within such a heterogeneous framework of farmed species and stunning and killing methods, both the World Organization for Animal Health (WOAH) and the European Food Safety Authority (EFSA) have provided recommendations and opinions, in order to safeguard the welfare of fish at the time of killing (12–20). Considering the main farmed species, WOAH suggested the following stunning/killing methods as humane: percussion stunning for carp and salmonids; spiking or coring for tuna; and electrical stunning for carp, eel, and salmonids (20). According to EFSA, new methods of stunning/killing are needed, since most of those commonly practiced in Europe do not allow many fish species to be humanely slaughtered (12–19).

In order to be considered humane, a method must involve killing the animal in a state of unconsciousness and insensibility. For this reason, major improvements are necessary regarding the evaluation of unconsciousness or death after the application of the method (12). This control is very difficult under field conditions, as demonstrations of stress or pain in fish are not obvious and complicated to detect. In fact, the best method for recognizing fish unconsciousness is the electroencephalogram (EEG) in a laboratory condition, with the observation of visual evoked responses (VERs) (12, 21–24), which is difficult to be used in the field. According to WOAH and EFSA, spontaneous behavior, responses to stimuli and reflexes, such as the loss of body and respiratory movement and the loss of the vestibulo-ocular reflex should be assessed. However, this assessment presents several technical and practical problems in the field due to the large number of slaughtered species and the lack of standard procedures to follow. Fish are slaughtered in groups while most of the indicators refer to an individual fish (25). The indicators have only been validated in the laboratory for a few species (12) and due to physiological and morphological differences among species, they are not always applicable in the same way. For the assessment of unconsciousness, a specific and adequate training of the operators, likewise Standard Operating Procedures (SOPs) to clarify how to perform it (e.g., number of fish checked/cage) are required.

Italy plays an important role on the European panorama, representing the third largest country in terms of aquaculture fish production (11) with around 55 thousand tonnes in the last 10 years (11, 26). Italy is the largest European producer of sturgeon (*Acipenser* spp.), the second of rainbow trout and catfish (*Ictalurus punctatus* and *Ameiurus melas*) and the third of European seabass, gilthead seabream, and European eel (*Anguilla anguilla*) (11, 26, 27). The farms currently operating to rear fish for human consumption are 558, mainly located in the north: Veneto (114 farms), Piedmont (70), Friuli-Venezia Giulia (69), Trentino-South Tyrol (59), and Lombardy (56),(28).

Currently, there is very little information on the practices used in Italy to protect the welfare of fish at the time of killing as there is no a specific national legislation for the protection of the fish at farm and at slaughter (9). The only available data is that collected in 2018 by the European Commission about the methods used to kill three farmed species (9). It is also not known if the effectiveness of the methods applied are evaluated, and what indicators are used. There are not national guidelines on fish welfare at slaughter but guidelines for fish welfare at slaughtering were recently issued by associations of fish farmers. However, they are generic, not mandatory and do not provide any SOPs on stunning or on the assessment of fish unconsciousness.

In this context, where the information available on what happens in the field is limited, it is of utmost importance to have data on the different realities present in Europe in order to produce applicable legislation without an excessive economic impact on the producers.

For these reasons, the aim of this study was to provide a detailed presentation of the current situation regarding fish welfare at the time of slaughtering in Europe’s third largest aquaculture producer, Italy. Using a questionnaire, we collected information on the fish-slaughter facilities currently operating on the Italian territory and on the practices used at the key moments of slaughter: the methods used to stun and kill fish and the indicators used to assess the unconsciousness. The assessment of the conformity of the procedures reported by the facilities was carried out in accordance with WOAH guidelines and EFSA recommendations on the protection of fish at slaughter (12–20).

## 2. Materials and methods

### 2.1. Data collection

The data were collected with the support of the Italian National Health System. The Italian Ministry of Health entrusted the Regional Competent Authorities to select an Official Veterinarian from the Regional and Local Health Units (LHU—ASL) responsible for safety and hygiene of food of animal origin for each Region. The selection was made based on their background and their expertise on slaughter as well as on their knowledge of the reality of aquaculture in their territory. The data collection was made through a questionnaire to fill with the information of the fish-slaughter facilities operating within their district. The completed questionnaires have been gathered in different moments as it has not been easy to register or verify the necessary data in all facilities simultaneously. The first questionnaire was collected in August 2022, while the last one was delivered by March 2023.

Official veterinarians were instructed on how to collect the data by means of an explanatory document prepared and disseminated by the Italian Ministry of Health. Support to the official veterinarians was given by the authors belonging to the CReNBA (Italian Reference Center for Animal Welfare—Istituto Zooprofilattico della Lombardia e dell’Emilia Romagna, Brescia) throughout the entire data collection period in order to clarify any possible doubt regarding how to fill in the questionnaire. The completed questionnaires were sent via email by each Italian region to the Italian Ministry of Health, which subsequently sent the questionnaire to the CReNBA for the analysis.

### 2.2. Development of the questionnaire

The questionnaire was developed by the CReNBA taking into account the structure and technique used in a veterinary questionnaire survey on welfare issues (29); the questions asked in a recent similar survey carried out in Brazil (30); and the generic recommendation of the European Regulation 1099/2009 on the protection of animals at time of killing. The targets of the questionnaire were the Italian Regional Competent Authorities, which sent it to their selected Official Veterinarians.

The questionnaire consists of an Excel document containing six open and closed questions, with the aim of collecting information about fish-slaughter facilities (Table 1). The first two questions related to the identification of the facilities, like their physical location (question 1) and their registration details (question 2). The third question was an open question on the annual processed fish volumes (question 3). This question was fundamental to be able to assess the volumes processed by the surveyed facilities in order to understand their relevance to the overall Italian aquaculture fish production. The following questionnaire question focused on the slaughtered species (question 4), with the possibility to select from seven fish species representing the main species farmed in Italy considering the most recent censuses of FEAP and FAO (26, 43). The last two questions regarded the stunning and/or killing methods (question 4) and indicators of unconsciousness applied (question 5), which had to be reported for each slaughtered species. The list of possible answers was built considering the main methods and indicators reported in literature as indicated in Table 1. The last three questions also allowing the veterinarians to add other possible options (open answer to fill in if necessary) in order to have the most accurate overview of the applied procedures.

**Table 1:** Areas and type of information requested in the questionnaire used for data collection in fish-slaughter facilities in Italy.

Questionnaire area	Type of information
Geographic area	Italian region
	Local Competent Authority (veterinary public health system)
Identification of the slaughter facility	Company name
	Address
	Official registration number (approval number)
What is the annual processing volume (tonnes)?	Open Answer
Which species are slaughtered?	Rainbow Trout (*Oncorhynchus mykiss*)
	European seabass (*Dicentrarchus labrax*)
	Gilthead seabream (*Sparus aurata*)
	Arctic char (*Salvelinus alpinus*)
	Common carp (*Cyprinus carpio*)
	European eel (*Anguilla anguilla*)
	Sturgeon (*Acipenser* spp.)
	Other species, please specify
What is the stunning/killing method used? (13–21, 31–37)	Percussion
	Electric in water bath
	Electric dry system
	Carbon monoxide (CO)
	Carbon dioxide (CO_2_)
	Asphyxia in ice/Thermal shock
	Asphyxia in air
	Other method, please specify
Which indicators are used to assess fish unconsciousness? (13–20, 24, 32, 38–42)	Breathing
	Eye movements
	Coordinated movement
	Response to stimuli
	Righting ability
	Other indicator, please specify

### 2.3. Data analysis

The results of the questionnaire survey were subjected to descriptive statistical analysis in order to provide the geographical distribution of the slaughtering facilities, the stunning/killing practices, and the indicators used for the unconsciousness assessment during slaughter. For a clearer presentation of the results, slaughtered fish species were divided into two groups: main species (i.e., fish species slaughtered in more than five facilities) and minor species (i.e., species slaughtered in less than five facilities).

## 3. Results

Fourteen Italian regions were confirmed to practice fish slaughtering in a total of 67 facilities: Veneto, Piedmont, Apulia, Lombardy, Friuli Venezia Giulia, Tuscany, Sardinia, Marche, Trentino-South Tyrol, Sicily, Liguria, Emilia Romagna, Lazio, and Campania. The Competent Authorities of the remaining six regions communicated the absence of fish-slaughter facilities on their territory.

**Figure 1:**
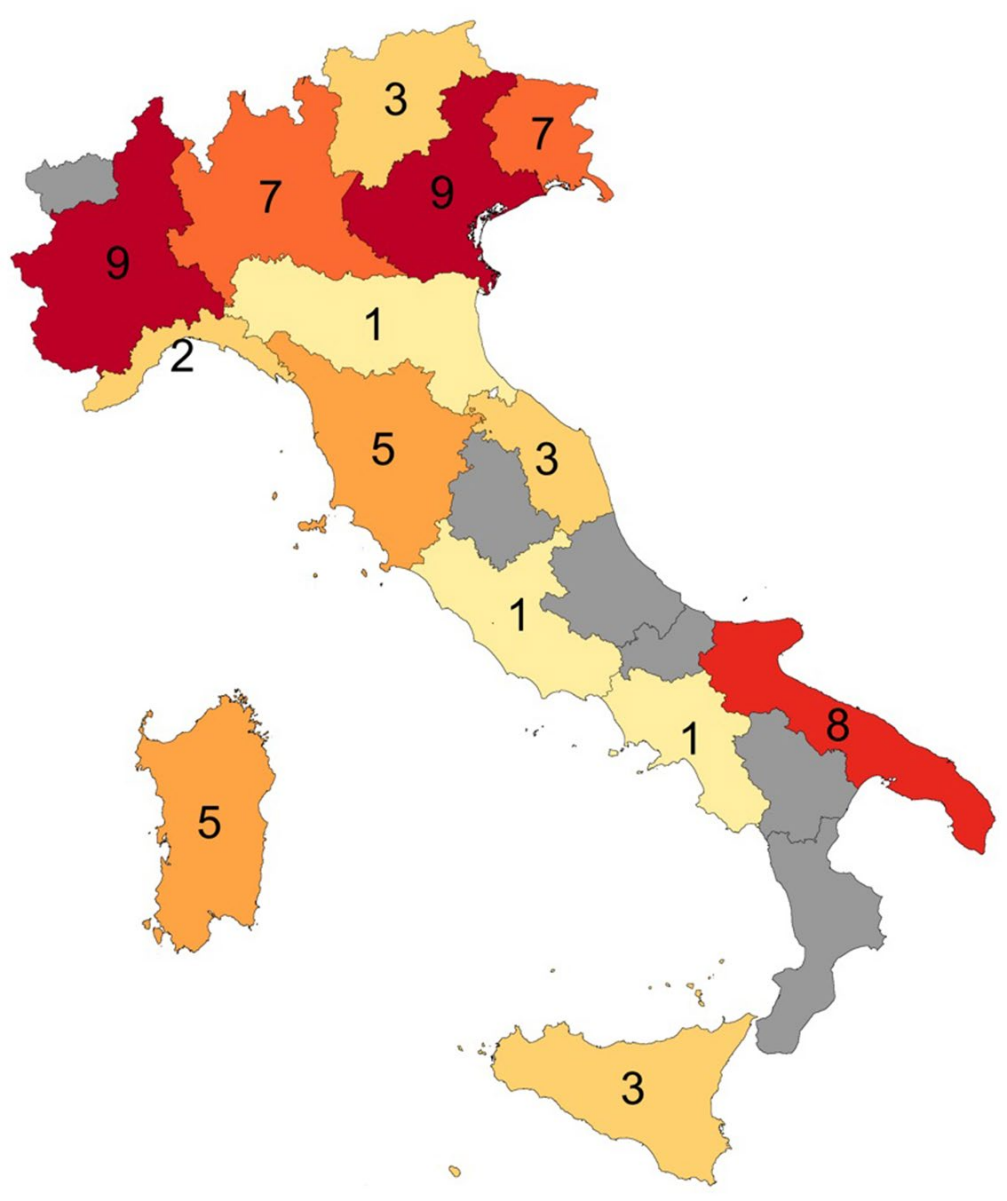
Distribution of the 64 fish-slaughter facilities in the Italian regions. In gray, the regions with no facilities.

Out of the 67 facilities, two slaughter facilities of rainbow trout (one in Veneto and one in Lombardy) and one of European seabass and gilthead seabream (in Sicily) were excluded from the analysis because they did not slaughter fish in the period used for collection of data. Therefore, the number of the facilities included for the data analysis on the fish stunning/killing methods in Italy was 64, where a total volume of 22,229 tonnes of fish was processed. The regions of Veneto, Piedmont, Apulia, Lombardy, and Friuli Venezia Giulia were the only ones which reported more than five fish-slaughter facilities in their territory (Figure 1). Marche, Veneto, Tuscany, Lombardy, and Lazio were the regions processing more than 1,500 tonnes of fish each (Table 2).

**Table 2:** Distribution of the different fish species slaughtered in the 64 Italian slaughtering facilities surveyed for data collection (*n* = number of fish-slaughter facilities in the region).

	Italian region														
	Veneto (*n* = 9)	Piedmont (*n* = 9)	Apulia (*n* = 8)	Lombardy (*n* = 7)	Friuli V.G. (*n* = 7)	Tuscany (*n* = 5)	Sardinia (*n* = 5)	Marche (*n* = 3)	Trentino ST (*n* = 3)	Sicily (*n* = 3)	Liguria (*n* = 2)	E. Romagna (*n* = 1)	Lazio (*n* = 1)	Campania (*n* = 1)	Total (*n* = 64)
	*5,559 t.*	*62 t.*	*1,266 t.*	*2,114 t.*	*1,121 t.*	*2,663 t*.	*752 t.*	*6,249 t.*	*61 t.*	*55 t.*	*438 t.*	*38 t.*	*1849 t.*	*2 t.*	*22,229 t.*
Rainbow trout	6	5	-	4	4	1	1	3	3	2	-	-	-	-	**29**
Seabass-seabream	-	-	7	-	3	3	4	-	-	-	2	-	1	1	**21**
Arctic char	2	4	-	1	2	-	-	2	3	-	-	-	-	-	**14**
European Eel	4	3	1	1	-	1	1	-	-	-	-	1	-	-	**12**
Sturgeon	3	3	-	4	-	-	-	-	-	-	-	1	-	-	**11**
Common carp	4	1	-	1	-	-	-	-	-	-	-	-	-	-	**6**
Brown trout	-	-	-	1	1	-	-	2	1	-	-	-	-	-	**5**
Meager	-	-	2	-	-	-	-	-	-	-	-	-	1	-	**4**
Trout perch	-	1	-	-	-	-	-	-	-	1	-	1	-	-	**3**
Hybrid strip. Bass	-	1	-	-	-	-	-	-	-	1	-	1	-	-	**3**
Royal perch	1	-	-	-	-	-	-	-	-	-	-	1	-	-	**2**
Gray mullet	-	-	-	-	-	-	2	-	-	-	-	-	-	-	**2**
Catfish	-	1	-	-	-	-	-	-	-	-	-	1	-	-	**2**
Lavaret	-	-	-	-	-	-	-	-	1	-	-	-	-	-	**1**
Danube salmon	-	-	-	-	-	-	-	-	1	-	-	-	-	-	**1**
Pikeperch	1	-	-	-	-	-	-	-	-	-	-	-	-	-	**1**
Tench	-	1	-	-	-	-	-	-	-	-	-	-	-	-	**1**
Greater amb.	-	-	-	-	-	1	-	-	-	-	-	-	-	-	**1**
Sargo	-	-	-	-	-	-	1	-	-	-	-	-	-	-	**1**
Turbot	-	-	-	-	-	-	-	-	-	-	-	-	-	1	**1**
															

The annual volume of fish processed for human consumption is reported by each region.

Fish slaughtering in Italian facilities involved 20 different species, and the 64% of the facilities processed more than one species. The main slaughtered fish species in Italy were rainbow trout (29/64), followed by European seabass and gilthead seabream (21/64), Arctic char (*Salvelinus alpinus*; 14/64), European eel (12/64), sturgeon (11/64), common carp (6/64), and brown trout (*Salmo trutta fario* L.; 5/64). The minor slaughtered fish species: meager (*Argyrosomus regius*), hybrid striped bass common called “persico-spigola” (*Morone chrysops* × *Morone saxatilis*), trout perch (*Micropterus salmoides*), gray mullet (*Mugil cephalus*), catfish (channel catfish—*Ictalurus punctatus* and black bullhead—*Ameiurus melas*), lavaret (*Coregonus lavaretus*), Danube salmon (*Hucho hucho*), pike-perch (*Sander lucioperca*), royal perch (*Perca fluviatilis*), turbot (*Psetta maxima*), sargo (*Diplodus sargus*), tench (*Tinca tinca*), and greater amberjack (*Seriola dumerili*; Table 2).

**Figure 2:**
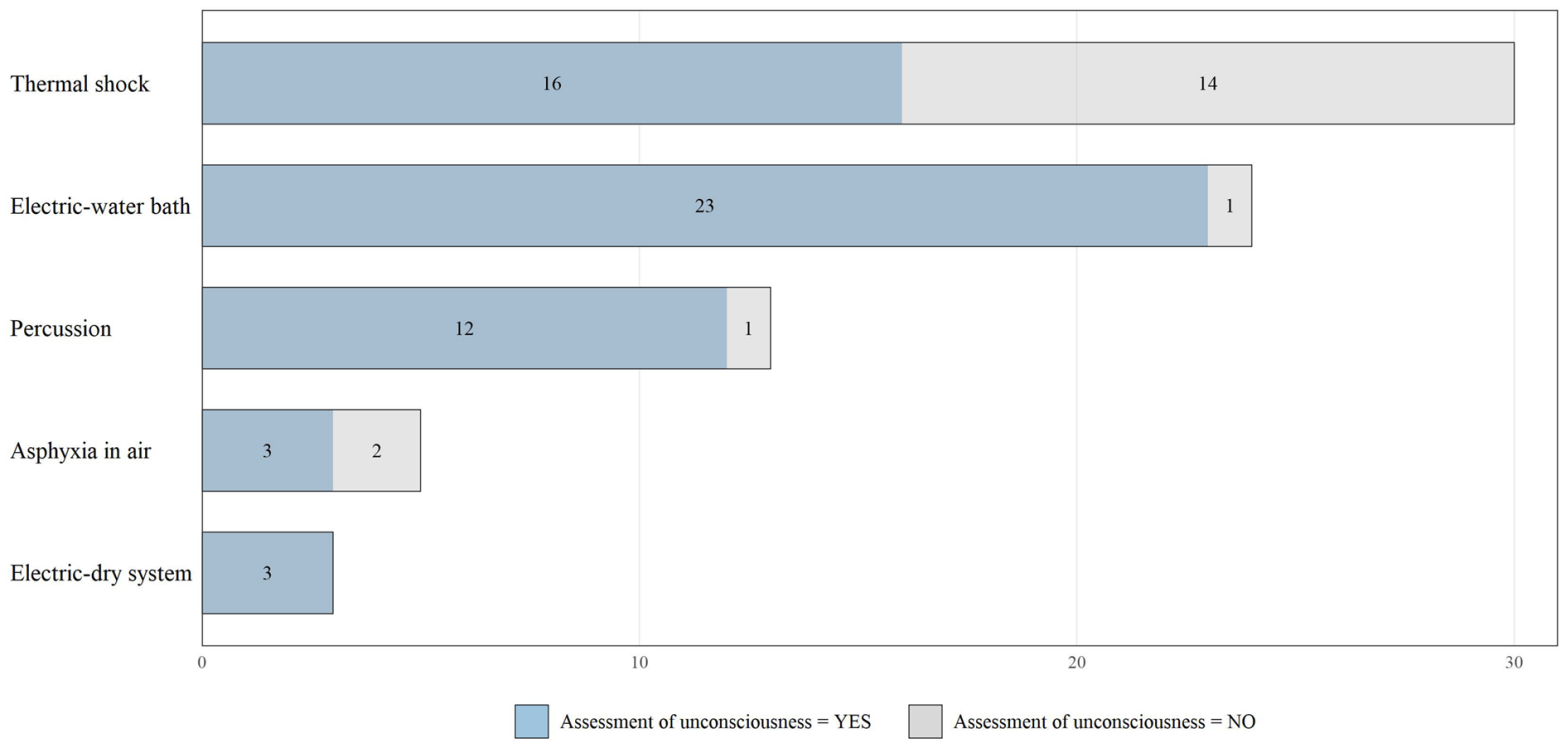
Distribution of different stunning/killing methods reported by the 64 fish-slaughter facilities surveyed, in association with the practice of assessing fish unconsciousness.

The majority of facilities (86%) practiced only one method, while a few used to practice more than one (14%). The most common methods applied were “asphyxia in ice/thermal shock” and “electric in water bath,” which were used in 30 and 24 of the facilities respectively, followed by “percussion” (13), “asphyxia in air” (5), and “electric dry system” (3) as shown in Figure 2. Two facilities did not perform any stunning or killing and sold live carps and eels directly to the consumer.

The different stunning/killing methods and the frequently used indicators to assess the effectiveness of the reported methods for the slaughtered species are shown in Tables 3, 4, respectively.

**Table 3:** Stunning/killing methods and indicators of consciousness used for the main slaughtered fish species in the surveyed fish-slaughter facilities.

		
	Stunning/killing methods						Indicators of unconsciousness				
	*Thermal shock*	*Electric water bath*	*Percussion*	*Asphyxia in air*	*Electric dry system*	Sold alive*	*Breathing*	*Eye movements*	*Coord. movements*	*Response to stimuli*	*Righting ability*
Rainbow trout (*n* = 29)	7	17	7	4	1	-	24	10	19	10	10
Seabass-seabream (*n* = 21)	20	-	-	1	-	-	4	1	6	2	1
Arctic char (*n* = 14)	2	10	3	1	-	-	13	6	10	6	7
European eel (*n* = 12)	3	8	-	1	-	2	5	1	9	6	2
Sturgeon (*n* = 11)	3	3	6	-	2		7	4	6	5	6
Common carp (*n* = 6)	2	4	-	1	-	1	5	1	2	4	2
Brown trout (*n* = 5)	-	4	1	-	-	-	5	2	3	2	1

^*^The practice “sold alive” is also reported.

**Table 4:** Stunning/killing methods and indicators of consciousness used for the minor slaughtered fish species in the surveyed fish-slaughter facilities.

		
	Stunning/killing methods					Indicators of unconsciousness				
	*Thermal shock*	*Electric water bath*	*Percussion*	*Asphyxia in air*	*Electric dry system*	*Breathing*	*Eye movements*	*Coord. movements*	*Response to stimuli*	*Righting ability*
Meager (*n* = 4)	3	-	-	-	-	1	1	1	-	-
Trout perch (*n* = 3)	3	-	-	-	-	3	3	2	3	3
Hybrid strip. Bass (*n* = 3)	3	-	-	-	-	3	3	2	3	3
Royal perch (*n* = 2)	2	1	-	-	-	2	1	1	1	1
Gray mullet (*n* = 2)	1	-	-	1	-	-	-	1	-	-
Catfish (*n* = 2)	2	-	-	-	-	2	2	1	2	2
Lavaret (*n* = 1)	-	-	1	-	-	1	1	1	-	1
Danube salmon (*n* = 1)	-	-	1	-	-	1	1	1	-	1
Pikeperch (*n* = 1)	1	1	-	-	-	1	-	-	-	-
Tench (*n* = 1)	-	1	-	-	-	-	-	1	1	-
Greater amb (*n* = 1)	1	-	-	-	-	-	-	-	-	-
Sargo (*n* = 1)	1	-	-	-	-	-	-	1	-	-
Turbot (*n* = 1)	1	-	-	-	-	1	-	1	1	-

**Figure 3:**
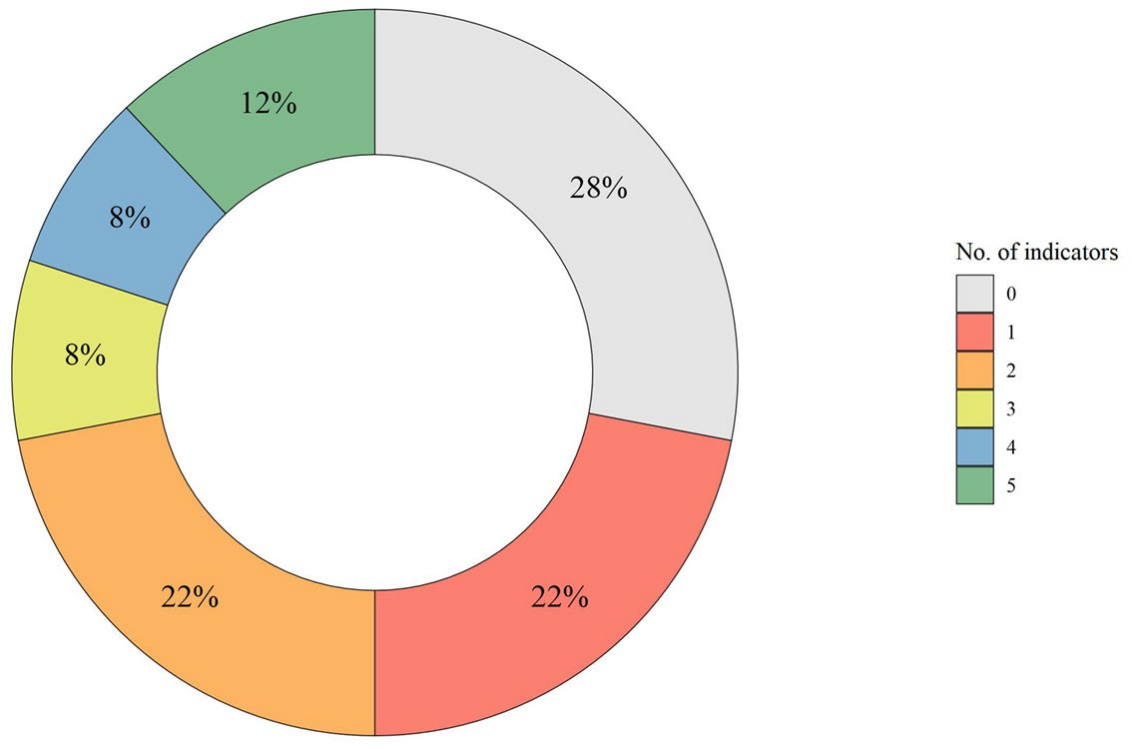
Distribution of the number of indicators considered for the assessment of fish unconsciousness during slaughter. The value 0 shows the number of fish-slaughter facilities that do not perform any assessment.

Looking in detail at the main species, data showed that thermal shock method was used in 95% of the cases for slaughtering of European seabass and gilthead seabream; the electric in water bath method was utilized in more than half of the facilities for rainbow trout, Arctic char, European eel, common carp, and brown trout; percussive method was practiced for sturgeon in more than half of the facilities. Also for minor species, the most used method is thermal shock while the electric in water bath, percussion and asphyxia were less practiced. Overall, the assessment of unconsciousness or death was carried out routinely in 45 of the surveyed facilities (72%). In details, for the main species, more than 90% of the facilities assessed unconsciousness or death, with the exception of European seabass and gilthead seabream which were assessed in only 28% of facilities. For minor species, the assessment was performed in 66% of facilities. The 50% of all facilities assessed the unconsciousness using more than one indicator (Figure 3). “Breathing” and “coordinated movements” were the indicators most frequently used (34 facilities), followed by “response to stimuli” (18), “righting ability” (15), and “eye movements” (14).

**Figure 4:**
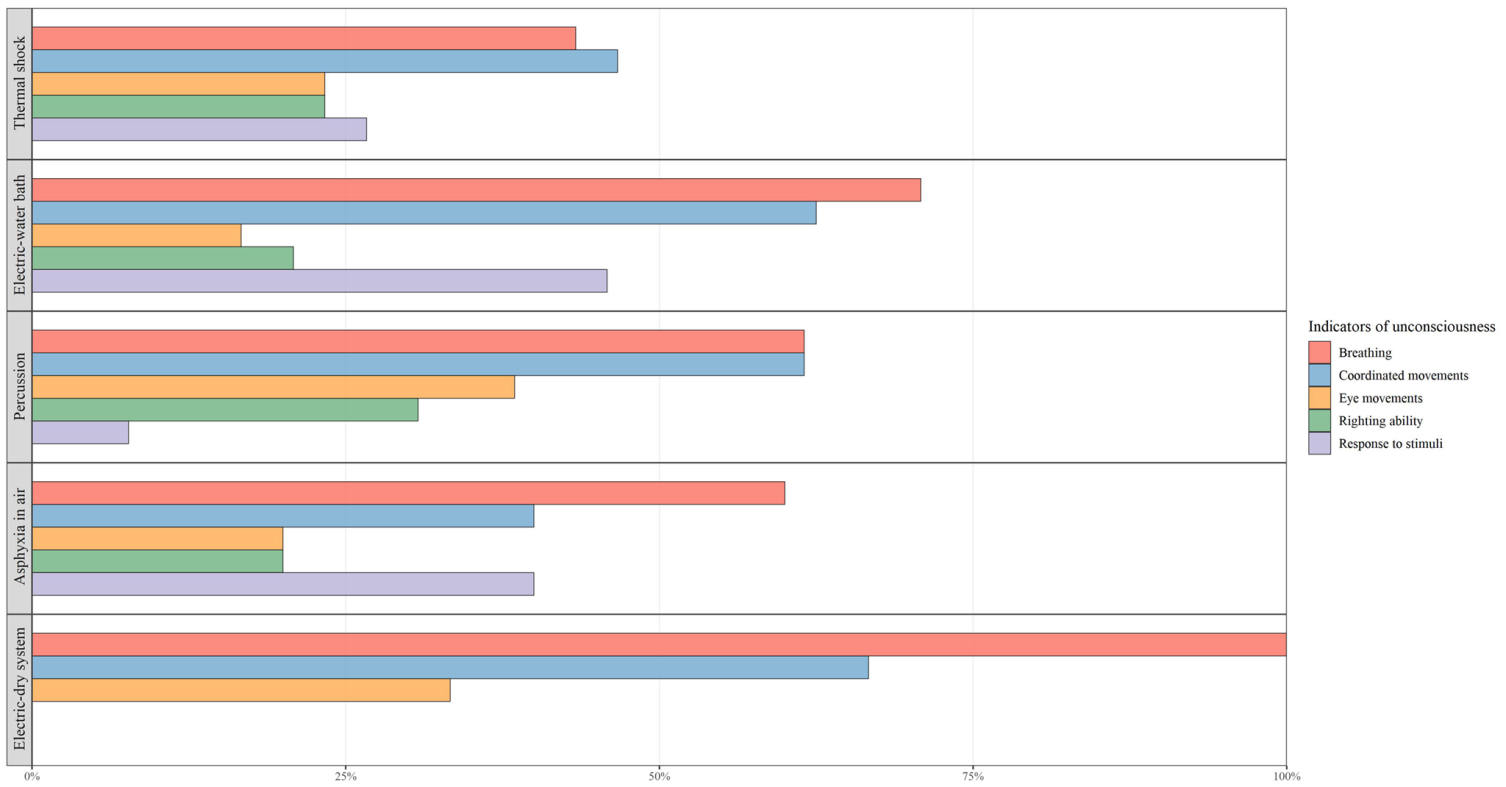
Indicators used by fish-slaughter facilities to assess unconsciousness according to the stunning/killing method used.

“Breathing” and “coordinated movements” were the most frequently assessed indicators for rainbow trout (24/29 and 19/29 respectively), Arctic char (13/14 and 10/14), European seabass and gilthead seabream (4/21 and 6/21), and brown trout (5/5 and 3/5). The indicator “response to external stimuli” is also assessed in about half of the facilities that slaughtered European eel (6/12) and common carp (4/6). For sturgeon “righting ability” is also assessed (6/11). Relating the stunning/killing methods to the assessment of unconsciousness, the electric and percussion methods are those for which the assessment of unconsciousness is practiced in more than 90% of cases in contrast to the ice and air asphyxia methods, for which the assessment is practiced around the 55% of the times (Figure 2). The distribution of the considered indicators used to assess fish unconsciousness differentiated by the methods used, is shown in Figure 4.

## 4. Discussion

### 4.1. General consideration on slaughter procedures

#### 4.1.1. Stunning/killing methods

The 14 Italian regions that participated in the survey and confirmed to practice fish slaughtering correspond to the regions with the highest presence of fish farms for human consumption in Italy (95% of all farms). In particular, the top five regions in terms of number of fish farms are Veneto, Piedmont, Friuli-Venezia Giulia, Trentino-South Tyrol, and Lombardy (28). Therefore, we can consider the collected data a significant representation of the current Italian situation of fish farmed and slaughtered for human consumption. As a matter of fact, considering the volumes processed by the 64 facilities, the results show that the data in this survey refer to the 41% of the total volume processed in Italy in 2022 (53,900 t.) (44). Consistent with production data (43), the results of the survey show that rainbow trout is the species most commonly slaughtered, followed by European seabass, gilthead seabream.

The survey also showed that the several facilities practiced slaughtering of more than one species, which could be a critical point for fish welfare, since WOAH (20) recommends that stun and slaughter facilities should be designed and constructed for one specific species or group of species in order not to compromise their welfare and not to cause injuries or stress.

A wide variety of methods were used in the surveyed facilities for the slaughtering of rainbow trout, Arctic char and sturgeon, whereas mainly one method was used for European seabass and gilthead seabream. According to the European legislation on the slaughter of farmed species (EC Reg. 1099/2009) and the recommendations of the WOAH and EFSA, animals must be subjected to a stunning process before being slaughtered in order to become unconscious and insensible. However, based on the results of the survey, in Italy, more than half of the fish-slaughter facilities (35/64) practiced the asphyxia in air or the thermal shock method. These procedures are considered non-humane methods of slaughtering fish by WOAH and EFSA. In fact, the asphyxia in air is considered only as a ‘killing’ method; the thermal shock does not stun the fish but produces only sedation, leading the conscious animal to death by asphyxia (12, 20). The remaining methods used in the Italian fish-slaughter facilities are generally recommended by the WOAH and EFSA as acceptable methods. They are namely the electrical methods and the percussion method, followed by gill-cutting, which, if carried out correctly, can induce a state of instantaneous unconsciousness before slaughtering (12). Specifically, the electrical method induces unconsciousness, but its efficacy can vary considerably according to the parameters set (mainly V, A, Hz and time of application) (12, 20). The details for these parameters were not collected in our study and need further investigations.

#### 4.1.2. Assessment of unconsciousness or death

One of the key aspects of evaluating the welfare of fish slaughtered in the field is to assess their state of unconsciousness or death after the application of the stunning/killing method. In our survey, there were no facilities that reported the assessment of unconsciousness or death by EEC, due to evident practical problems, whereas the majority of the surveyed facilities (72%) used the evaluation of the reflexes, spontaneous behavior or response to external stimuli, as recommended by EFSA and WOAH (12, 20). Indeed, there was a big difference in the frequency of this assessment, depending on the fish species slaughtered and method used (Figure 4). The low number of facilities (28%) assessing unconsciousness or death on European seabass and gilthead seabream was actually striking. This data negatively influences the overall result, since unconsciousness was assessed in almost all facilities for all the other main species. Probably, this result is related to the problem of the assessment of the individual fish unconsciousness, which is particularly difficult when the production volumes are high. For this reason, it is recommended to have group indicators available, as suggested by Noble et al. in their handbooks (45, 46).

Most Italian facilities carried out this assessment using the indicators “breathing” and “coordinated movements” which are probably more easily to check compared to eye movement assessment on an individual fish. However, assessment by breathing and body movements can be ineffective or unreliable indicators (47). In fact, as previously reported, asphyxia in ice and incorrectly performed electrical methods are recognized to paralyze fish, thus making it unable to behaviorally express pain (12). There are currently no behavioral indicators that can fully differentiate paralysis from unconsciousness or death (12). In the absence of alternatives, the assessment of a combination of different indicators can improve the evaluation (48, 49), as it occurred in half of the surveyed facilities (Figure 3).

### 4.2. Species-specific consideration on slaughter procedures

Since one of the objectives of the survey was to relate the collected slaughtered practices with the available species-specific recommendations (Table 5) among the species slaughtered in Italy, only those covered by these recommendations were taken into account for this purpose, i.e., rainbow trout, common carp, European eel, turbot, European seabass, gilthead seabream and, in some aspects, also the other species belonging to the *Salmonidae* family (Arctic char, brown trout, Danube salmon and lavaret). For the other eleven slaughtered species, the general recommendations on welfare at slaughter of the WOAH and EFSA (12, 20) were used.

**Table 5:** General and specific WOAH (20) and EFSA recommendations (12–19) on stunning/killing methods of farmed fish for human consumption.

	Percussion		Electric		Spiking or coring		Asphyxia in air or ice	
					Free bullet		CO2	
	*WOAH*	*EFSA*	*WOAH*	*EFSA*	*WOAH*	*EFSA*	*WOAH*	*EFSA*
General considerations	✓	✓	✓	✓	✓	✓	✗	✗
Rainbow trout	✓	✓	✓	✓	✗	✗	✗	✗
Eel	✗	*	✓	✓	✗	✗	✗	✗
Carp	✓	*	✓	*	✗	✗	✗	✗
Seabream—Seabass	n/a	n/a	n/a	*	n/a	✗	n/a	✗
Turbot	n/a	*	n/a	*	n/a	✗	n/a	✗
Atlantic Salmon	✓	✓	✓	✓	✗	✗	✗	✗
Other salmonids	✓	✓	✓	✓	✗	✗	✗	✗
Tuna	✗	✗	✗	✗	✓	✓	✗	✗

✓, recommended/acceptable method; ✗, not recommended method; ^*^, recommended/acceptable method which requires further studies on this species; n/a., species-specific recommendations not available.

#### 4.2.1. Rainbow trout

Based on the results of the present survey, trout was the species slaughtered with the highest variety of slaughtering methods in Italian facilities, which is consistent with available data about the other major European producers of trout (France and Denmark) and more generally whole of Europe (9, 16). The most commonly used stunning/killing method was the electric one, like in Denmark (9), which, if carried out correctly, satisfies the recommendations of both WOAH and EFSA (16, 20). This result confirms the use of the electric practice on trout in Italy, as previously reported (9). The second most practiced method was percussion, which is the most used method in France (9). If correctly done within 10 s from the moment the fish is pulled out of the water, percussion is also considered a humane method for trout (16). As in other European countries, asphyxia in ice and air were also practiced, although they should be avoided, since they do not induce effective loss of consciousness (9, 12, 20, 50).

The assessment of trout unconsciousness was mainly carried out using “breathing” and “coordinated movements” as recommended by WOAH (20). According to EFSA (16), however, these indicators are considered acceptable as indicative of unconsciousness but are not really robust or validated in laboratory conditions. As a matter of fact, although some authors have recently stated that loss of respiratory movement could be related to unconsciousness (24), others found no clear relationship between loss of ventilation and brain failure in rainbow trout under laboratory conditions (47). Thus, we can conclude that the assessment of unconsciousness/death for trout in Italy can be considered to be correctly carried out in the majority of the fish slaughter facilities.

#### 4.2.2. European eel and common carp

Different slaughter methods have been reported for eel in our survey in Italy, as in other European countries (15), with the electric method the most widely used. This method is among those recommended by WOAH (20) for this species and has recently been introduced by the first two major European producers, i.e., the Netherlands and Germany (31). Non-humane procedures such as live evisceration or baths in ammonium salts (15), reported to be carried out in other European countries, were not practiced in Italian slaughtering facilities. Nevertheless, both eel and carp can be sold alive to the consumer in Italy (practice previously reported by EFSA in Europe only for carp) (19) which poses possible risks to the welfare of the fish at the time of killing (19). However, this practice was not widespread in Italy compared to northern European countries (19) and was confined only to two regions (Puglia and Veneto). The electric method was the main method practiced in Italy for carp, as in the top two European carp producers, Germany and Poland (9). This method satisfies WOAH recommendations, whereas EFSA highlighted some critical points on the methods and required more investigations in order to be able to express itself more accurately (19). However, recent studies by Daskalova et al. (51) have pointed out that the electrical method can be considered an acceptable method for carp, with a low impact on the welfare of the slaughtered carps. In particular, the use of electrical stunning alone could not make the carp unconscious for a long time, as demonstrated by quick VER recovery after stunning (VER were recorded already at 30 s post stunning) (32). Interestingly, in the field study conducted by Retter et al. (32) in Germany, the majority of farms used a combination of electrical stunning immediately followed by manual percussive stunning (59%). Under this condition, 92.6% of stunned carps displayed no behavioral indicators of consciousness and significantly fewer injuries related to mishits compared to sole percussive stunning. Thus, using a combination of electrical stunning and percussion could be a better option for this species, as the use of the singular methods could not be exhaustive in inducing unconsciousness under field conditions. In our survey, for both eel and carp, killing by ice or air asphyxia were used. However, this can pose risks for fish welfare since carp can survive up to 5 h in apnea (52) and eel, due to its peculiarity of being able to partially breathe with its skin, can survive even for days (12).

The assessment of unconsciousness for carps and eels was carried out mainly using “breathing,” “coordinated movements,” and “response to stimuli,” that are among those recommended by WOAH (20). However, this assessment suggests caution in its application because of the physiological peculiarities of these species: e.g. resistance to breathing out of the water (described above) and as for the eel, the ability to move the body even when the brain is dead. In fact, for eel, in the surveyed facilities, the assessment was carried out taking into account the fish movements and breathing, which are considered not reliable indicators by EFSA (15).

#### 4.2.3. European seabass, gilthead seabream, and turbot

For seabass, seabream, and turbot, asphyxia ice in was confirmed as the most widely used practice in Italy for slaughtering. This method is not accepted by WOAH (20), although it represents the most common method used also by the other main European producers, Spain and Greece (9). According to EFSA, an alternative slaughter method is requested for these species (14, 17). In fact, thermal shock has been shown to cause immobility and paralysis of fish: seabream remains conscious for up to 5 min after immersion in water and ice (53) and turbot even up to 90 min (54). The use of anesthetics (i.e., clove oil) (55, 56) or the diffusion of gases (i.e., carbon dioxide or nitrogen) (35, 57) in the stunning tank allow fish to reach faster unconsciousness and death than ice slurry application alone. For the same reason, research testing of electrical stunning reached promising results in seabream and seabass. This method, followed by a thermal shock, has recently been introduced in Europe in some seabass and seabream farms on an experimental basis (9). Recent studies have shown that the electrical method can be a valid alternative for the slaughter of turbot also (58). The introduction of this method also in Italian facilities should be considered if experimental data confirm an improvement of the current slaughter conditions for these species.

In the majority of the surveyed facilities, the slaughter of these fish took place without the assessment of unconsciousness or death. When assessed, the main indicators used namely “breathing” and “coordinated movements,” have been found in some studies to be not reliable and not robust indicators, even more so in regard to the main method applied, which causes paralysis (12, 17).

#### 4.2.4. Salmonids (different from rainbow trout)

For Arctic char, brown trout, Danube salmon and lavaret, the main methods currently used in the surveyed Italian facilities are among those reported by WOAH specific recommendations on stunning/killing methods for species of the *Salmonidae* family (20), i.e., percussion and electric.

For these species, fish unconsciousness was monitored in almost all facilities, mainly by assessing the respiration and coordinated movements, among the indicators recommended by WOAH (20). To the knowledge of the authors, no scientific study or species-specific opinions/guidelines are available about the reliability of these indicators for these species.

Certain recommendations contained in EFSA’s opinions on Atlantic salmon and trout can generally be extended to Arctic char and brown trout and other salmonids. However, caution should be exercised when using these recommendations as there are different species within the family that react differently to stunning and killing methods (e.g., Arctic char has been shown to be strongly resistant to electricity).

#### 4.2.5. Other species

In the surveyed facilities, most of the species in this group were slaughtered using methods considered non-humane, which should be replaced (12, 20). Only asphyxia (in ice and air) was used for meager, trout perch, hybrid striped bass, gray mullet, catfish, greater amberjack, and sargo. For royal perch and pikeperch, electrical stunning was also utilized and should be preferred from a welfare point of view (12, 20).

Sturgeon and tench were the only species of this group to be slaughtered mainly in accordance with the general recommendations of WOAH and EFSA (12, 20) for appropriate slaughter, i.e., with percussion and electric methods. However, it should be highlighted that these are very different species both in terms of size and behavior, thus there is an evident criticality when following the general guidelines for the analysis. For sturgeon, the mechanical or electrical methods perfectly fall within the generically recommended methods by WOAH and EFSA (12, 20). However, stunning procedures can considerably differ from facility to facility. Based on data collected by the survey, some facilities used rubber hammers, others used steel hammers, sometimes inside, some other outside the aquatic environment. In one case, the use of the sheep-specific stunner was also reported. It is evident how these methods have numerous practical critical issues, including dependence on the subjectivity of the operator and on his/her ability, training or state of fatigue. Last, but not least, while slaughtering methods should be species-specific, the use of the same methods and the same working conditions for different species, especially when minor species are involved, is a critical point. For the sturgeon in particular, that is a species so different from the other farmed and slaughtered species, there is a general lack of specific recommendation. Recently, Williot et al. (59) reviewing the existing Siberian Sturgeon farming procedures in relation to their welfare, highlighted that there is no published study focused on slaughter of Sturgeon. Finally, while most of surveyed facilities monitored unconsciousness in fish belonging to this group by the indicators recommended by WOAH (20), species-specific and validated indicators are lacking for these species.

## 5. Conclusion

This is the first study providing data about the systems used for stunning and killing fish and the methods used to evaluate the unconsciousness in Italian fish-slaughter facilities. In view of the revision of the European legislation about welfare of farmed animals and in the absence of field data about fish, extensive information about the systems currently used for slaughtering is pivotal for all the stakeholders in order to have an overview of fish welfare at slaughter.

Based on the collected information, methods considered non-humane are still widely used for the slaughtering of fish in Italy, especially for sea bass, sea bream, and the less slaughtered species, whereas for the other species, slaughtering mainly follow the WOAH recommendations. The lack of scientific data and validated indicators make it difficult to obtain a clear picture of the welfare condition at slaughter for many species. On the other hand, as for rainbow trout, i.e., the major farmed fish in Italy, both the used stunning/killing methods and the indicators of unconsciousness are consistent with species-specific recommendations about welfare at slaughter.

In conclusion, more studies are necessary to clearly identify the best species-specific methods to protect fish at slaughter and the best indicators to be used to assess unconsciousness and death. Similarly, it is pivotal to gather more data on the feasibility and efficacy of these methods and indicators in field conditions as well as their economic impact.

## Data availability statement

The raw data supporting the conclusions of this article will be made available by the authors, without undue reservation.

## Author contributions

SR and GC: conceptualization and funding acquisition. SR, GC, AT, TD, DB, FB, and VL: methodology. GC, SR, CT, and AB: formal analysis and investigation. GC, SR, and AT: writing—original draft preparation. GC, SR, AT, AM, CT, and VL: writing—review and editing. SR and AT: supervision. All authors contributed to the article and approved the submitted version.
